# Tumor Necrosis Factor (TNF)-Alpha Inhibitors in Hidradenitis Suppurativa: A Systematic Review of Inflammatory Markers and Alternative Monitoring Strategies

**DOI:** 10.7759/cureus.97849

**Published:** 2025-11-26

**Authors:** Rupanshu R, Russaal S Mann, Isha Chopra, Abdullah Kilic, Ayushi Saxena, Bilal Khan, Safeera Khan

**Affiliations:** 1 Internal Medicine, St. Martinus University Faculty of Medicine, Willemstad, CUW; 2 Internal Medicine, Vardhman Mahavir Medical College and Safdarjung Hospital, New Delhi, IND; 3 Anesthesia, Mahatma Gandhi Medical College and Hospital, Jaipur, IND; 4 Internal Medicine, Hackensack University Medical Center, Montclair, USA; 5 Medicine, California Institute of Behavioral Neurosciences & Psychology, Fairfield, USA; 6 Health Sciences, Queen's University, Kingston, CAN; 7 Family Medicine, Michener Institute, University Health Network, Toronto, CAN

**Keywords:** acne inversa, adalimumab, c-reactive protein(crp), erythrocyte sedimentation rate (esr), hidradenitis suppurativa (hs), inflammatory markers, tnf-alpha blocker, tnf-alpha inhibitors

## Abstract

Hidradenitis suppurativa (HS) is a long-standing inflammatory skin condition with inflammatory nodules, abscesses, and sinus tracts. Tumor necrosis factor-alpha (TNF-alpha) inhibitors like Adalimumab are one of the first-line therapies approved for use in moderate-to-severe HS, but despite that, there is no consensus on what objective biomarkers can be employed to monitor treatment efficacy. This systematic review aims to explain the utility of C-reactive protein (CRP) and erythrocyte sedimentation rate (ESR) as reliable indicators of treatment response to HS. Our objective was to determine if, in patients with HS, treatment with TNF-alpha inhibitors (e.g., adalimumab) leads to a significant change in systemic inflammatory markers like CRP and ESR. We followed Preferred Reporting Items for Systematic Reviews and Meta-Analyses (PRISMA) 2020 guidelines. Subsequently, a systematic search was conducted across databases like PubMed/MEDLINE, PMC, ScienceDirect, Cochrane Library, and Clinical Trials, focusing on studies published within the last five years. Search terms included "hidradenitis suppurativa," "adalimumab," and "infliximab." Included studies underwent rigorous quality appraisal with appropriate quality assessment tools, finally resulting in the analysis of only the relevant articles.

The evidence confirms that adalimumab therapy leads to a statistically significant reduction in CRP levels, mainly due to its systemic anti-inflammatory effect. However, the clinical value of CRP is questionable, as it is susceptible to confounding by obesity and metabolic syndrome, which are also highly prevalent comorbidities found in HS patients. In contrast, ESR is also proposed as a theoretically superior biomarker, one of the reasons being that it has less interference from these confounders. Our review highlighted a significant lack of prospective data evaluating ESR and CRP changes in response to TNF-alpha inhibitor therapy. A prominent trend was also noticed in recent clinical trials within the past five years to prioritize clinical checkpoints, such as the Hidradenitis Suppurativa Clinical Response (HiSCR), over serological markers.

Overall, our systematic review showed that CRP is not a perfect biomarker for monitoring individual treatment response, which is in part due to various confounding factors, such as obesity affecting its level. ESR represents a promising but as yet unvalidated alternative. Currently, the most useful strategy may be a hybrid approach that integrates clinical scores (e.g., HiSCR, International Hidradenitis Suppurativa Severity Score System [IHS4]) with the simultaneous use of inflammatory markers. Our review highlights a critical need for future research to evaluate and compare CRP and ESR to establish their definitive roles in guiding HS therapy.

## Introduction and background

The prevalence of hidradenitis suppurativa is approximately 1% worldwide, with some regional variations [1]. HS (Acne Inversa), also known as Verneuil's disease, is a long-standing inflammatory skin disorder characterized by the presence of painful nodules, abscesses, and sinus tracts, most likely located in the axillae and groin, but also found in the genitoanal and inframammary regions. While HS commonly presents in the third decade of life, its onset has also been described in postmenopausal women and even in children [2,3].

HS is frequently associated with comorbidities such as metabolic syndrome, inflammatory arthritis, obesity, and inflammatory bowel disease as well, reflecting its systemic inflammatory nature [1]. Among these risk factors, obesity is the factor that is strongly linked to HS, and given the increasing prevalence of obesity, it is equally important to analyze different treatment options [4]. Even though the pathogenesis of HS is not fully understood, it is generally thought to result from a multitude of factors such as endocrine, familial, environmental, and microbial [3]. Given the inflammatory background of the disease, current treatment focuses on reducing inflammation and includes drug therapy or combined drug and surgical therapy in more advanced stages, which can be measured by the Hurley staging system [1]. One category of anti-inflammatory therapies approved for moderate to severe HS is TNF-alpha inhibitors such as adalimumab, as well as interleukin-17 (IL-17) inhibitors such as secukinumab and bimekizumab. These therapies are usually reserved for difficult-to-control HS, which was previously treated mainly with oral antibiotics [1]. Antibiotics are still employed mainly for mild HS to control the microbial load and limit complications of the disease [3]. Table 1 describes the Hurley staging system.

**Table 1:** Hurley staging system Cicatrization: Process of forming a scar at the site of a healing wound

Hurley stage	Description
1	Presence of abscesses without any sinus tracts or cicatrization
2	Recurrent abscesses with sinus tract formation and cicatrization
3	Diffuse or near-diffuse involvement multiple interconnected tracts and abscesses across the entire area

Recent advancements in biologics and immunomodulators have led to significantly improved patients’ quality of life while at the same time reducing the need for surgical interventions [3]. Early interventions are really essential in order to prevent irreversible skin damage and scarring, as well as for adequate control of symptoms such as pain and reduction of extracutaneous comorbidities, all of which require early diagnosis and an interdisciplinary and personalized approach [1]. Over the last decade, evidence for new treatment options for moderate-to-severe HS has been growing significantly. Therefore, understanding which inflammatory markers can be used to monitor treatment response, prevent relapses, and improve quality of life is crucial. This systematic review focuses on one specific treatment option for HS: TNF-alpha inhibitors, mainly adalimumab. Although evidence for other treatment classes, such as interleukin (IL)-17 inhibitors, IL-1 inhibitors, and Janus kinase inhibitors, has been growing in recent years, adalimumab remains the mainstay of treatment due to its high efficacy and several clinical studies [3]. Currently, only adalimumab, secukinumab, and bimekizumab are the FDA-approved treatment options for moderate-to-severe HS, with the choice between them guided mainly by patient comorbidities and preferences [2].

This systematic review focuses on whether inflammatory markers such as CRP and ESR can reliably monitor disease severity and whether treatment with adalimumab produces clinically relevant changes in these markers that correlate with outcomes. In summary, this review tries to address the following question: In patients with HS, does treatment with TNF-alpha inhibitors (e.g., adalimumab) produce significant changes in systemic inflammatory markers (e.g., CRP, ESR)?

Due to the increasing incidence of HS worldwide, it is imperative to establish strong and reliable indicators for treatment response. We aim to analyze whether these inflammatory markers are effective for this purpose or whether alternative biomarkers are needed for a better understanding of disease severity.

**Figure 1:**
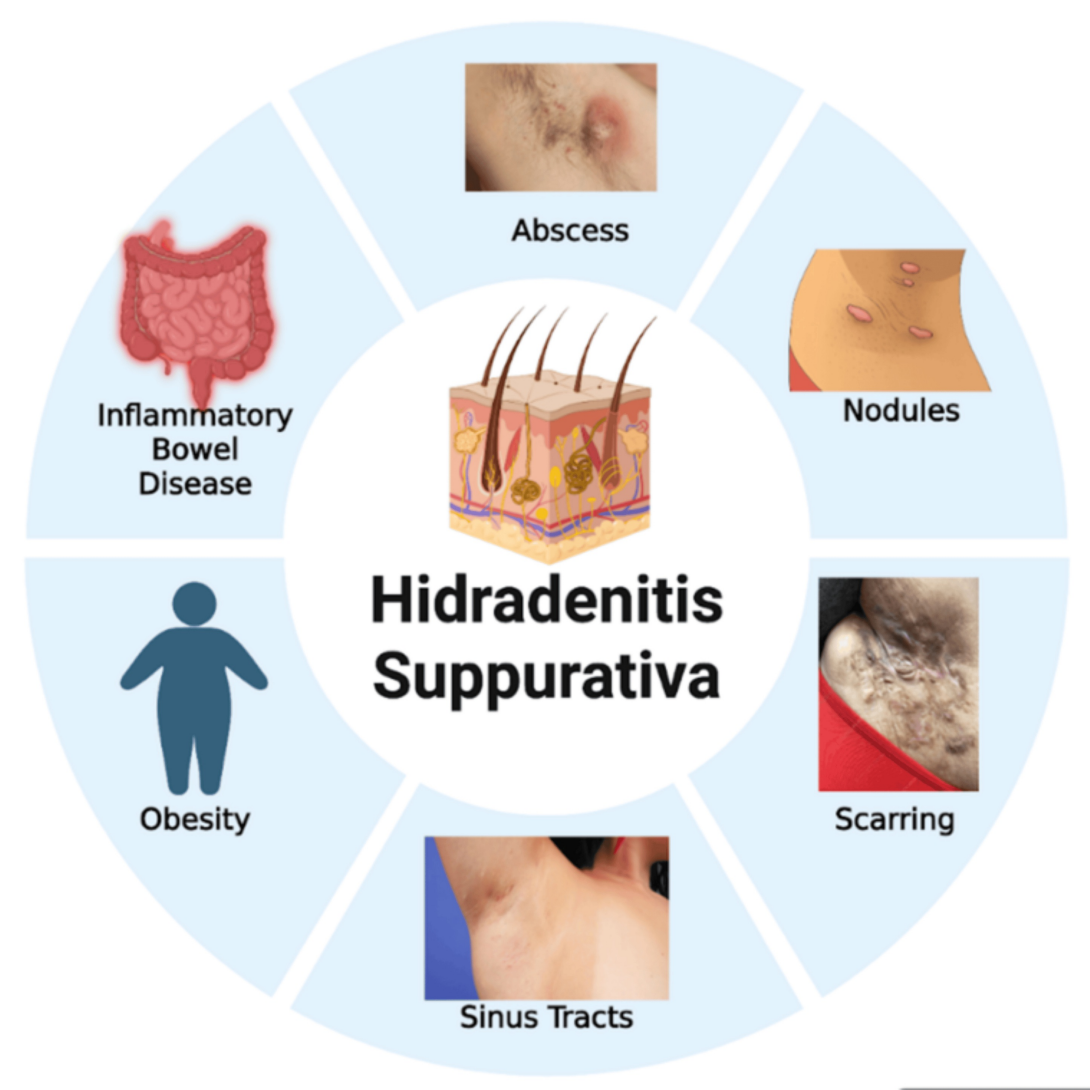
Manifestation and associations of HS Image credits: Rupanshu Created in Biorender [5]

Figure 1 explains both the association and the manifestation of hidradenitis suppurativa.

## Review

Methods

**Figure 2:**
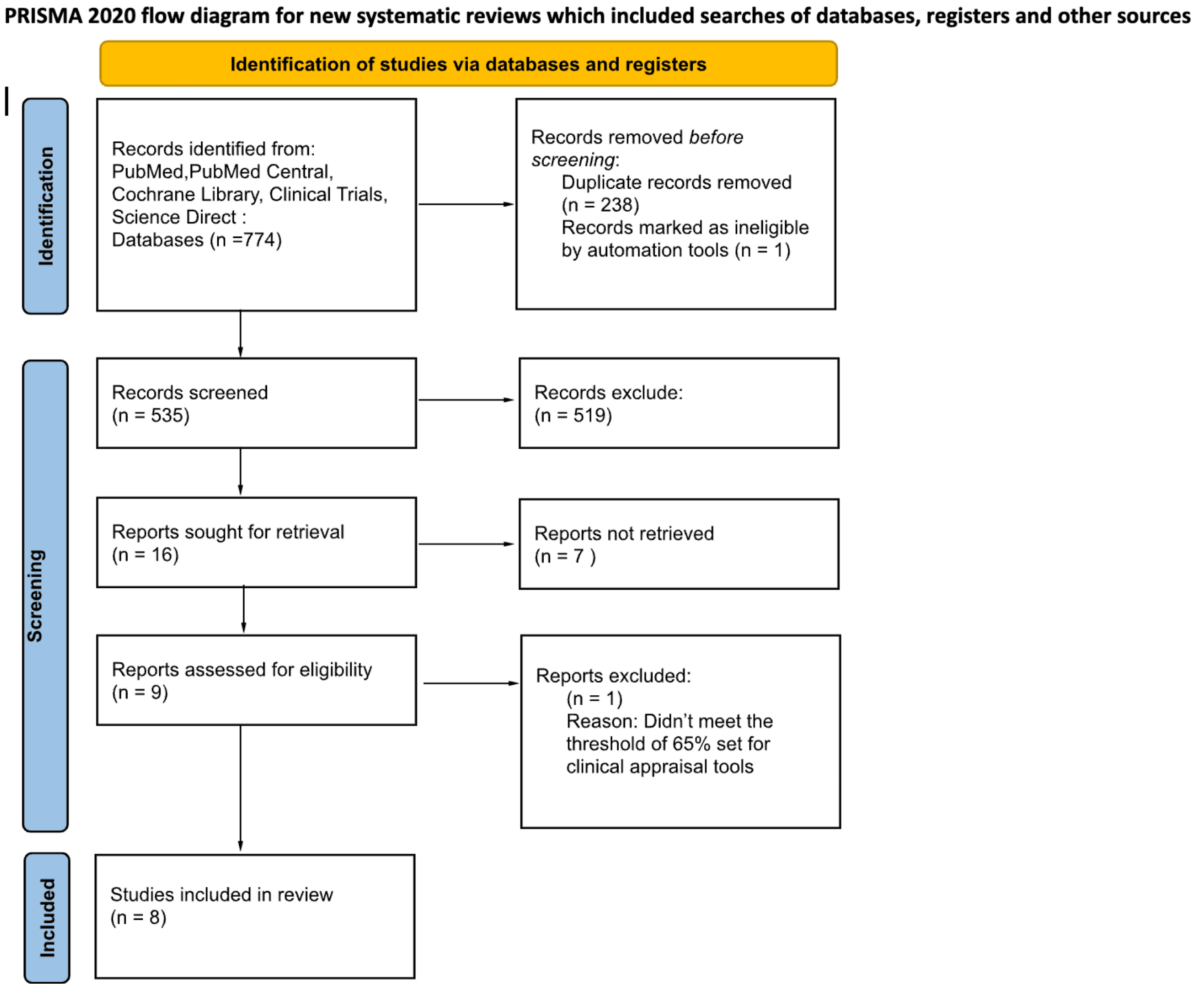
PRISMA flowchart PRISMA: Preferred Reporting Items for Systematic Reviews and Meta-Analyses [6]

This systematic review was conducted in accordance with the Preferred Reporting Items for Systematic Reviews and Meta-Analyses (PRISMA) 2020 guidelines [6]. A complete PRISMA flow diagram is shown in Figure 2.

Search strategy and selection criteria

A comprehensive literature search was performed in order to identify the relevant studies by systematically searching several databases, including PubMed/MEDLINE, PubMed Central (PMC), ScienceDirect, the Cochrane Library, and Clinical Trials. The search strategies were customized for each separate database by using various combinations of the keywords, such as "hidradenitis suppurativa," "acne inversa," "infliximab," and "adalimumab." In the PubMed/MEDLINE database, a Medical Subject Headings (MeSH) strategy of ("Hidradenitis Suppurativa"[MeSH] OR "Acne Inversa") AND ("adalimumab"[MeSH] OR "infliximab"[MeSH] OR "Tumor Necrosis Factor-alpha/antagonists and inhibitors"[MeSH]) was employed. This strategy was further refined using an advanced search, also including title and abstract in the database for ("hidradenitis suppurativa"[Title/Abstract]) AND (infliximab[Title/Abstract] OR adalimumab [Title/Abstract]). In Clinical Trials and the Cochrane Library, the search was conducted using ("Hidradenitis Suppurativa" OR "Acne Inversa") AND (adalimumab OR infliximab). For both PMC and ScienceDirect, ("Hidradenitis Suppurativa" OR "Acne Inversa") AND (adalimumab OR infliximab) AND ("C-reactive protein" OR "ESR"), the search strategy was used. The outcomes of the search strategies are shown in Table 2.

**Table 2:** The databases used and the identified number of papers for each database MeSH: Medical Subject Headings, PMC: PubMed Central, ESR: Erythrocyte sedimentation rate

SEARCH STRATEGY	DATABASE USED	Number of papers identified
"hidradenitis suppurativa" AND INFLIXIMAB AND ADALIMUMAB	PubMed	93
("Hidradenitis Suppurativa"[Mesh] OR "Acne Inversa") AND ("adalimumab"[Mesh] OR "infliximab"[Mesh] OR "Tumor Necrosis Factor-alpha/antagonists and inhibitors"[Mesh])	PubMed (MeSH)	427
("hidradenitis suppurativa"[Title/Abstract]) AND (INFLIXIMAB[Title/Abstract] AND ADALIMUMAB[Title/Abstract])	PubMed (Advanced)	71
("Hidradenitis Suppurativa" OR "Acne Inversa") AND (adalimumab OR infliximab)	Clinical Trials	22
("Hidradenitis Suppurativa" OR "Acne Inversa") AND (adalimumab OR infliximab)	Cochrane	1
("Hidradenitis Suppurativa" OR "Acne Inversa") AND (adalimumab OR infliximab) AND C-reactive protein, ESR	PMC	156
("Hidradenitis Suppurativa" OR "Acne Inversa") AND (adalimumab OR infliximab) AND C-reactive protein, ESR	Science Direct	4

Inclusion and exclusion criteria

Our study focused on the most recent literature published within the past five years, and only those articles were further accessed. We specifically selected articles written in English or those with available full-text translations into the English language. In order to ensure more relevance to our research, we only included studies that involved human participants, and any animal studies were disregarded. Articles were then checked for the availability of full text, and articles where the full text could not be accessed were excluded from our review. Gray literature was also not included in the analyzed articles. The exclusion and inclusion criteria are listed in Table 3.

**Table 3:** Inclusion and exclusion criteria HS: Hidradenitis Suppurativa, TNF: Tumor Necrosis Factor

INCLUSION CRITERIA:	EXCLUSION CRITERIA:
Papers focussing on systemic TNF-alpha inhibitors including Adalimumab or Infliximab or on only one of them.	Papers focussing on other biologics like etanercept.
Papers focussing on all age groups.	Papers focusing on topical or intralesional therapies only.
Papers with focus on only Humans as subjects.	Patients with a diagnosis of HS that is not confirmed.
Papers published in the last 5 years.	Papers focusing on animal studies.

Quality assessment and selection process

The articles were imported into Zotero, and duplicate papers were then merged and removed. After that, each article underwent screening first based on its title, and then, if we were still not sure, we accessed its abstract. The articles pertinent to relevant information were then subjected to a thorough evaluation of their full texts, and only those deemed appropriate were included in the assessment. Inclusion and exclusion criteria were carefully applied at the end as well, resulting in the final selection of articles.

The shortlisted articles thereafter underwent a rigorous quality assessment using appropriate quality appraisal tools tailored to different types of study design. The Newcastle-Ottawa Scale (NOS) was mainly employed to evaluate the quality of observational studies, including prospective and observational cohort studies and non-randomized control trials, while the Assessment of Multiple Systematic Reviews (AMSTAR) tool was utilized to assess the quality of systematic reviews. Only studies that met the criteria set by the quality appraisal were included in the systematic review, ensuring that only high-quality studies were considered for analysis. A score of 6 or above was considered a high-quality study and included in the systematic review, which corresponds to the 65% threshold.

The quality appraisal for each study is shown in the following Tables 4-6.

**Table 4:** AMSTAR checklist for systematic reviews AMSTAR: Assessment of multiple systematic reviews; RoB: Risk of bias; PICO: Population, intervention, control, outcome Criteria sourced from Shea et al. [7]

AMSTAR checklist	Systematic review
	Chiang et al. [3]
PICO included in the research question and inclusion criteria?	No
Explicit statements to show review methods were established prior to conduct of the review? Any justification of significant deviations from the protocol?	No
Did the review authors explain their selection of the study designs for inclusion in the review?	Yes
Utilization of a comprehensive literature search strategy?	Yes
Was study selection performed in duplicate by review authors?	Yes
Was data extraction performed in duplicate by review authors?	Yes
Was a list of excluded studies with justifications provided by the review authors?	Yes
Were the included studies described in adequate detail?	partial yes
Was a satisfactory technique used for RoB assessment in individual studies included in the review?	No
Did the review authors report on the funding sources for the included studies in the review?	Yes
Usage of appropriate methods for statistical combination of results if a meta-analysis was performed?	No meta-analysis conducted
If meta-analysis was performed, was the potential impact of RoB assessed with regard to individual studies?	No meta-analysis conducted
Did the review authors account for RoB in individual studies when interpreting/discussing the results of the review?	No
A satisfactory explanation for any heterogeneity observed in the results of the review given?	Yes
If they performed quantitative synthesis did the review authors carry out an adequate investigation of publication bias and discuss its impact on the results of the review?	No meta-analysis conducted
Were any potential sources of conflict of interest, including funding they received for conducting the review reported?	No

**Table 5:** Newcastle-Ottawa Scale (NOS) quality assessment Criteria sourced from Wells et al. [13]

Study ID	Representative of the exposed cohort	Selection of external control	Ascertainment of exposure	Outcome of interest not present at the start	Main factor	Additional factor	Assessment of outcomes	Sufficient follow-up time	Adequacy of follow-up	Total (9/9)
Hayashi et al. 52-week analysis [8]	*	0	*	*	0	0	*	*	*	6/9
Hayashi et al. 12-week interim analysis [9]	*	0	*	*	0	0	*	*	0	5/9
Gulliver et al. [10]	*	0	*	*	*	0	*	*	*	7/9
Hafner et al. [11]	*	0	*	*	*	0	*	*	*	7/9
Williams et al. [12]	*	0	*	*	*	0	*	*	*	7/9

**Table 6:** Cochrane bias table for quality appraisal Criteria sourced from Sterne et al. [15]

Study ID	Randomization	Allocation concealment	Incomplete outcome data	Selective outcome reporting	Other biases
Lu et al. [14]	High	Unclear	Low	Low	High

A study summary of all the findings, including the study type, biomarker(s) used, and key findings, is recorded in the following Table 7.

**Table 7:** Summary of studies on inflammatory biomarkers (ESR/CRP) in hidradenitis suppurativa CRP: C-reactive protein, ESR: Erythrocyte sedimentation rate, hs-CRP: High-sensitivity CRP, HiSCR: Hidradenitis Suppurativa Clinical response, HS-PGA: HS-Physician Global Assessment

Study	Study Type	Biomarker(s) Tested	Key Results/Findings
Chiang and Alhusayen [3]	Review Article	ESR and CRP	Summarized all the existing evidence and knowledge, noting that ESR and CRP are increased at baseline in active HS patients and discussed their use as objective markers to monitor inflammation and response to biologic therapies.
Hayashi et al. [8]	Postmarketing Surveillance Study	CRP	At the end of week 52, mean CRP levels in patients treated with adalimumab significantly decreased from 1.63 mg/dL at baseline to 0.54 mg/dL.
Gulliver et al. (SOLACE Study) [10]	Real-world Observational Cohort Study	CRP	Treatment with adalimumab was effective in reducing median CRP levels from baseline.
Hafner et al. (HARMONY Study) [11]	Real-world Observational Cohort Study	CRP	A statistically significant decrease in CRP levels was noted in patients treated with adalimumab.
Williams et al. [12]	Retrospective Cohort Study	None; HS-PGA and HiSCR	Mainly focused on clinical improvement in patients treated with adalimumab dose escalation from 40mg/week to 80mg/week and in these patients a corresponding reduction in CRP.
Lu et al. [14]	Systematic Review and Meta-analysis	CRP	Meta analysis of RCT showed adalimumab led to statistically significant decrease in CRP levels.
Gibson et al. [16]	Prospective Cohort Study	ESR and CRP	Suggested ESR may be a better biomarker than CRP for predicting disease severity.
Kimball et al. [17]	Post Hoc Analysis of Clinical Trial Data(PIONEER I & II)	hs-CRP	Patients who achieved the primary clinical endpoint via HiSCR, showed significant reductions in hs-CRP compared to those who did not achieve the endpoint.

Results

A total of 774 articles were found employing different search strategies. Out of these 774 articles, 591 were from PubMed using various strategies, including advanced and MeSH; 156 were from PMC; and the remaining 22 were from Clinical Trials, one from the Cochrane Library, and the remaining four from Science Direct. After the removal of 238 duplicates and one paper that was retracted according to automation tools, in the next step, screening was done according to the inclusion and exclusion criteria of 535 remaining articles. Out of them, 519 papers were excluded. Consequently, 16 papers were sought for retrieval, and after checking for the availability of full texts and filtering through titles and abstracts, we were left with nine papers. Of these articles, one article was removed as it didn’t meet the threshold set for quality appraisal. Subsequently, based on the intervention and outcome to be studied in our systematic review, we were left with eight papers.

Discussion

Hidradenitis suppurativa, if not controlled at an early stage, has been shown to be of a relapsing nature and negatively affect patients’ quality of life. Therefore, it is imperative that there should be ways of monitoring treatment response to HS, especially for the most commonly employed treatment for HS, i.e., TNF-alpha inhibitors, including but not limited to adalimumab [3]. This essential rationale for investigating and employing systemic, serological biomarkers can offer an objective system of complementing traditional clinical assessments and providing a more complete picture of disease severity and, at the same time, the physiological impact of therapeutic interventions like adalimumab.

**Figure 3:**
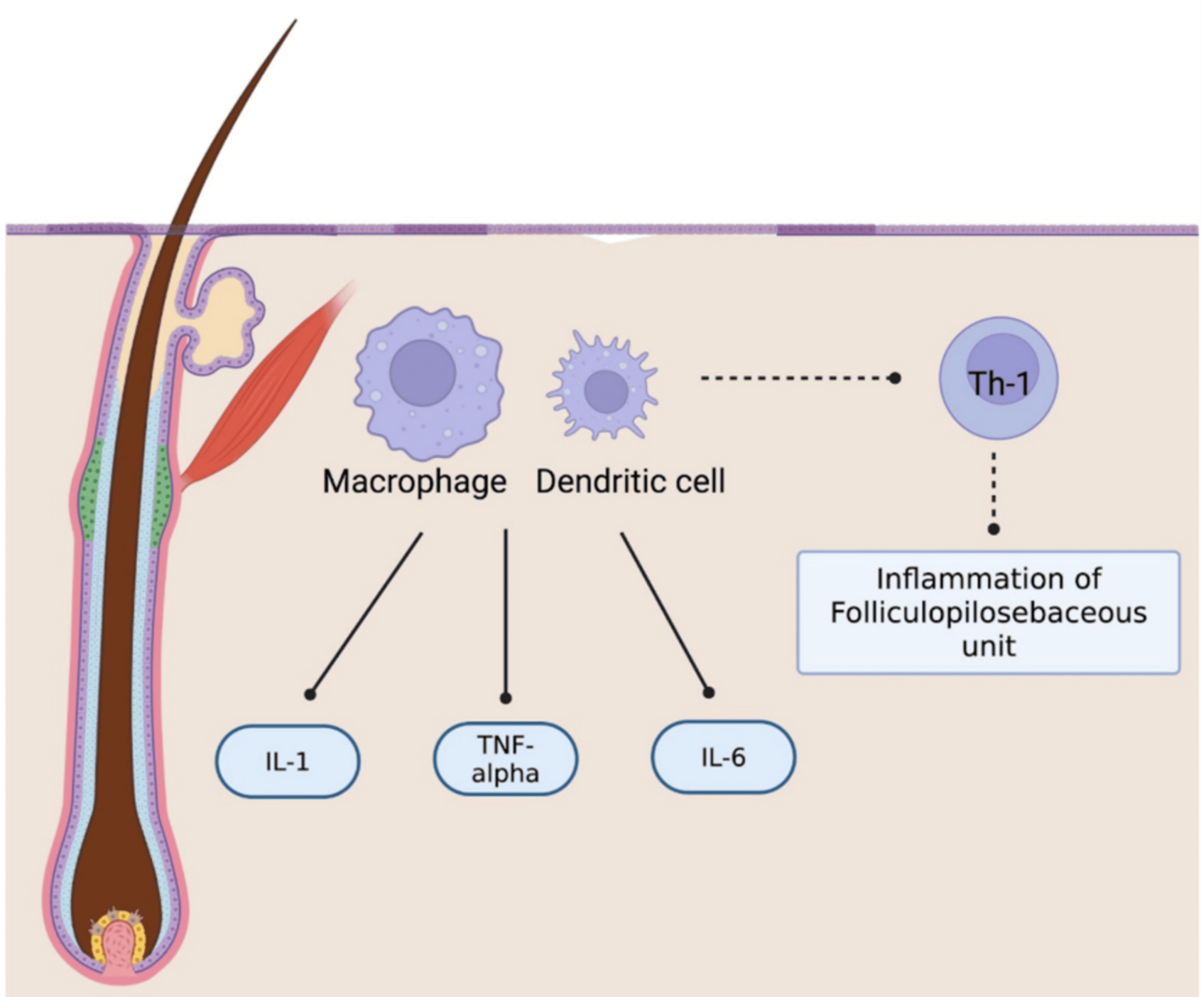
HS pathogenesis Th-1: T-helper 1 cells, TNF-alpha: Tumor necrosis factor alpha Created in Biorender Inspired by the Molinelli et al. article on the pathogenesis of HS [18].

Figure 3 illustrates the possible pathophysiology of hidradenitis suppurativa and the key role of TNF-alpha, IL-1, IL-6, and Th1 cells.

Evidence for CRP Reduction

The use of tumor necrosis factor-alpha (TNF-alpha) inhibitors, namely adalimumab, has significantly advanced the management of hidradenitis suppurativa, but the optimal biomarkers to monitor systemic inflammation and therapeutic response remain debated to this day. Real-world post-marketing surveillance (PMS) studies from Japan for one year have consistently shown that treatment with adalimumab results in a significant reduction in CRP levels [8]. The mean CRP level decreased from 1.8 mg/dL at baseline to 1.1 mg/dL at week 12, a reduction that was sustained through week 52, and this also aligned with clinical improvement in lesion counts of HS and quality-of-life (QoL) indices for the patient [8]. This PMS study also demonstrated that adalimumab was well tolerated regardless of patient demographics and characteristics [8]. All of these findings strongly support CRP as a responsive marker of systemic inflammation in HS under adalimumab therapy. But despite these data, CRP may not be an ideal marker in HS. 

CRP as a Negative Prognostic Indicator

A review of other studies has revealed that CRP may not be used as a good prognostic indicator due to its susceptibility to confounding from obesity and metabolic syndrome, both of which are prevalent in this patient population, as we discussed in the introduction section [3]. Obesity is strongly associated with HS, and if CRP can change due to these factors, how do we even know what is affecting its levels? Other real-world studies, such as Gulliver et al. SOLACE and Hafner et al. HARMONY did not even report CRP as a primary outcome measure, suggesting limited clinical relevance and unclear significance [10,11]. SOLACE, a real-world study of one year, also strongly supported the use of IHS4 (International Hidradenitis Suppurativa Severity Score System) to monitor the treatment instead of any of the inflammatory markers [10]. Tables 8, 9 describe how the IHS4 system is used to classify HS into mild, moderate, and severe types.

**Table 8:** International hidradenitis suppurativa severity scoring system (IHS4) N: Inflammatory Nodules, A: Abscesses, T: Draining Tunnels

IHS4 Scoring			
Lesion Type	Symbol	Weight	Formula Component
Inflammatory nodules	N	×1	N
Abscesses	A	×2	2×A
Draining tunnels	T	×4	4×T

**Table 9:** Hidradenitis suppurativa classification according to IHS4 IHS4: International Hidradenitis Suppurativa Severity Scoring System

Severity	Score Range
Mild	≤ 3
Moderate	4–10
Severe	≥ 11

ESR Might Be a Better Alternative

While CRP is a commonly used inflammatory marker for HS, a commentary by Gibson et al. proposes that ESR may be a more reliable and, therefore, preferred biomarker for HS disease severity [16].

Rationale for ESR

ESR is not actually a direct measure of inflammation but an indirect one [16]. It actually reflects the rate at which red blood cells aggregate due to the presence of plasma proteins like fibrinogen and immunoglobulins, which make the erythrocytes agglutinate together [16]. The high burden of inflammation in HS also causes a release of proteins that promote this aggregation, thus increasing the ESR [16]. Of note, ESR can also be independently elevated by several hematologic abnormalities that can be associated with HS, including anemia of chronic disease and thrombocytosis. CRP is elevated in response to cytokines in an inflammatory state, whereas ESR can be increased in response to multiple factors related to HS, including anemia and inflammation [16]. Therefore, this argument is not based on a direct comparison of post-treatment changes, but it is based on a fundamental analysis of the distinct pathophysiological factors that influence each marker [16]. 

In this context, erythrocyte sedimentation rate has been proposed as a potentially superior biomarker for monitoring HS-related systemic inflammation. Gibson et al. also argue that ESR may be better at reflecting the chronic inflammatory activity in HS, given its dependence on fibrinogen and its relative independence from adiposity compared to CRP [16]. It was also mentioned that ESR can remain elevated for days to even weeks, while CRP has a much shorter half-life and normalizes within days [16]. If ESR, unlike CRP, won't be affected by obesity or metabolic syndrome, then it can be a better alternative to our problem. However, prospective data evaluating ESR changes specifically during TNF-alpha inhibitor therapy are lacking, leaving its role as a treatment-response marker inconclusive for now [16]. Table 10 summarizes the discussion about the two inflammatory markers.

**Table 10:** Comparison of inflammatory biomarkers in hidradenitis suppurativa Summary of findings from Hayashi et al. [8] and Gibson et al. [16]

Biomarker	Pathogenesis	Advantages	Disadvantages
C-Reactive Protein (CRP)	Synthesized in response to cytokines in an inflammatory state.	Level is significantly reduced by adalimumab treatment, confirming a systemic effect.	High baseline levels may predict a poor clinical response to adalimumab. Can be affected by genetic polymorphisms and autoantibodies.
Erythrocyte Sedimentation Rate (ESR)	Reflects erythrocyte aggregation due to plasma proteins eg. fibrinogen, immunoglobulins	May be a more reliable indicator of overall disease severity. Reflects a more holistic disease burden, including anemia and chronic protein changes. Not affected by genetic polymorphisms or autoantibodies that can influence CRP.	The provided articles do not contain data on ESR reduction following adalimumab treatment.

A systematic review by Lu et al. did not include CRP as a marker of interest in their pooled analysis but included HiSCR, Modified Sartorius Score, and DLQI (Dermatology Life Quality Index) clinical assessments instead [14]. In this systematic review, including over 1000 patients, weekly adalimumab resulted in significant improvement in DLQI and modified sartorius score [14]. Also, a comprehensive analysis by Gibson & Kimball (2021) commentary reveals a critical finding, as the article does not contain or discuss any data related to the serological inflammatory markers CRP or ESR in the context of adalimumab treatment for hidradenitis suppurativa [19]. In this article, titled "Refining how we measure as we test," the decision to completely ignore these laboratory markers of systemic inflammation might represent a powerful, implicit argument. This deliberate omission might suggest that, in the authors' view, a "refined" approach to measuring HS must look beyond these traditional serological tests and instead focus on structural damage with draining tunnels as the key indicator of chronic, destructive disease and patient-reported burden, like the Quality-of-Life index, as the key points of discussion [19]. They also mentioned that with advances in our knowledge of HS, it is time we moved into an era that allows more head-to-head comparison with much longer primary endpoints [19]. So next, we will discuss what these scores and tools are that are employed in many of the studies for HS treatment response, and what they measure.

What to Measure in Clinics and Studies

Some other currently available clinical measures for assessing HS severity include the Hurley stage (Stages 1-3), the modified Sartorius score, and the HS Physician’s Global Assessment (HS-PGA); however, none of these measures have been validated in depth regarding patient impact and their clinical relevance [17]. Also, HiSCR is a tool that has also been used to assess the efficacy of treatment in a clinical setting to achieve hidradenitis suppurativa response [17]. This score is associated with significant improvement in clinical as well as patient-reported outcomes from the post hoc analysis of pooled data from the PIONEER I and II trials [17]. The use of HiSCR as a measure of treatment response is what ultimately led to the approval by the FDA of adalimumab for treating moderate-severe HS [17]. HiSCR is mainly employed as a practical measure and is based on a number of easily recognizable clinical signs, including inflammatory abscesses, nodules, and draining fistulas and sinus tracts [17]. IHS4, which stands for the International Hidradenitis Suppurativa Severity Score System, is also a validated tool used to quantitatively assess the severity of hidradenitis suppurativa. Because IHS4 employs the same clinical signs as abscesses, nodules, and draining fistulae/tracts as HiSCR, these two tools can be easily incorporated into a clinical practice [17]. Therefore, clinical outcome measures, including the HiSCR, IHS4, patient-reported pain and quality-of-life indices, and DLQI, remain the primary validated endpoints in HS trials and clinical practice [10,11]. HS-PGA, along with patient-reported symptoms, was also used to monitor treatment response when comparing different doses of adalimumab therapy in a retrospective cohort study by Williams et al., in which patients had a median follow-up time of 11.2 months to 13.2 months [12]. It was reported that with 80 mg/week of adalimumab therapy, HS-PGA had a point reduction, HiSCR was achieved in all the patients, and all patients reported improvement in symptoms with reduced pain and drainage of the lesions [12].

Given current data, a hybrid approach can be considered, meaning to (i) use HiSCR/IHS4 and pain/DLQI as primaries and (ii) track CRP on adalimumab and add ESR where feasible. Future trials should prospectively co-measure CRP, ESR, and selective cytokines to validate thresholds. 

Table 11 depicts what different strategies that can be used to evaluate the treatment response.

**Table 11:** Purpose, recommended measures, and their rationale in HS HiSCR: Hidradenitis Suppurativa Clinical Response, IHS4: International Hidradenitis Suppurativa Severity Score System, DLQI: Dermatology Life Quality Index, CRP: C-reactive Protein, ESR: Erythrocyte Sedimentation Rate Summary of findings inferred from Gibson et al. [16,19] and Kimball et al. [17]

Purpose	Recommended Measures	Why
Primary response	HiSCR, IHS4, lesion counts, pain, DLQI	Validated as well as treatment-sensitive
Systemic inflammation (routine)	CRP (especially on adalimumab), ESR	ESR may better reflect systemic inflammation in HS

Limitations

This discussion is also subject to several limitations. First, although CRP reduction with adalimumab is supported by real-world post-marketing surveillance data from Japan by Hayashi et al. in 2023 [8], such findings may not be generalizable to all populations due to ethnic, genetic, and healthcare system differences. Second, although ESR has been proposed as a superior biomarker for systemic inflammation, as it is not confounded by obesity and other factors in HS by Gibson et al., there are still not enough studies that directly evaluate its dynamics under TNF-alpha inhibitor therapy in patients [16]. This leaves its role as a treatment-response marker speculative rather than evidence-based, needing more thorough analysis before we draw any conclusions.

Additionally, like we discussed, CRP itself is confounded by obesity, metabolic syndrome [3], all of which are highly prevalent in HS, which complicates the interpretation of treatment-related changes. Similarly, ESR is influenced by age, anemia, and chronic conditions, limiting its specificity for HS. Finally, publication bias may exist, as studies with negative biomarker results may remain unpublished, thereby skewing the evidence base in one direction. Also, despite our best efforts, we could not recover any randomized controlled trials for our review, which would have proven to be excellent supplementary data.

## Conclusions

In conclusion, treatment with the TNF-alpha inhibitor adalimumab demonstrably leads to a significant decrease in C-reactive protein levels in patients with HS, which confirms its systemic anti-inflammatory effect as validated by many studies we discussed in our article. However, the clinical utility of CRP is complex, and data are lacking, with some studies not even reporting CRP as an outcome measure. At the same time, a compelling case can also be made for ESR as potentially a more comprehensive marker for HS, as it reflects a broader spectrum of the disease's systemic consequences without being affected by confounders like obesity. 

Although adalimumab has proven to be an effective treatment, the choice of biomarker for monitoring its effect requires careful consideration. Further research is needed to directly investigate and compare these inflammatory markers in patients with HS undergoing treatment with TNF-alpha inhibitors. There needs to be a pragmatic monitoring framework with CRP and ESR in parallel when available, particularly in obese patients, where CRP interpretation is confounded. Future trials should prospectively measure CRP, ESR, and other cytokine biomarkers in parallel to define their relative thresholds and clarify their role alongside clinical scores like IHS4 and HiSCR in guiding TNF-alpha inhibitor therapy in HS.
